# Bone Marrow Transplantation 1957-2019

**DOI:** 10.3389/fimmu.2019.01246

**Published:** 2019-06-05

**Authors:** Elizabeth Simpson, Francesco Dazzi

**Affiliations:** ^1^Division of Immunology & Inflammation, Department of Medicine, Imperial College London, London, United Kingdom; ^2^Division of Cancer Studies, King's College London, London, United Kingdom

**Keywords:** transplantation, histocompatibility, graft-vs.-host, graft-vs.-tumour, immunosuppression

## Abstract

Clinical bone marrow transplantation started in 1957 at a time when remarkably little was known about hematopoietic stems cells, immune responses to transplants or the identity of transplant antigens. This review will delineate the substantial increase in knowledge about these three areas gained between then and 1992 when the Ceppellini School course on Bone Marrow Transplantation was held, along with the progress made in clinical application, as well as the stumbling blocks that remained to be overcome by further research to advance knowledge. It will outline the significant progress made between 1992 and the present year, 2019, and the remaining problems.

## Introduction

**Figure 1:**
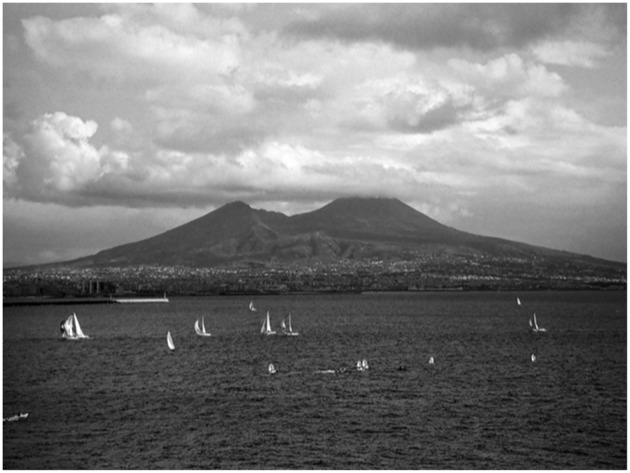
Naples, where Professor Serafino Zappacosta founded the Scuola Superiore d'Immunologia Ruggero Ceppellini in 1991. Image: Vera Maone, used with permission.

The Scuola Superiore d'Immunologia Ruggero Ceppellini (Ceppellini School) was founded in 1991 in Naples by Professor Serafino Zappacosta, to honor the memory and achievements of Professor Ruggero Ceppellini, a giant in the field of HLA genetics, who led an approach to addressing complex scientific questions through national and international collaboration (Figure 1). The Ceppellini school takes this forward by promoting contact and collaboration through residential post-graduate level courses led by international faculty for early career basic scientists and clinicians from advanced and developing regions and countries. It has accelerated Immunology education and influenced the evolution of other international schools of immunology. This issue of *Frontiers in Immunology* is a celebration of the achievements of the school and a tribute to Professor Serafino Zappacosta, Professor of Immunology at the “Federico II” University of Naples, who aimed to create in Southern Italy a pole of attraction for those pursuing immunological studies, and to promote interaction among the scientific and medical communities at the national and international level.

## Review of the Bone Marrow Transplantation Course

In 1992 Bone Marrow Transplantation was the subject of the inaugural course of the Ceppellini School. This topic brought into focus both genetics and immunology, the areas to which Ceppellini's research on hematological disorders and the human major histocompatibility complex, HLA, was pivotal.

This review of bone marrow/hematological stem cell transplantation will focus on how contributions to the 1992 Ceppellini School course on Bone Marrow Transplantation provide a mid-way marker point in the six decades following 1957 when Donnall Thomas first reported on six patients given bone marrow transplants to restore hemopoiesis following ablation by radiation or drug toxicity (1). He was encouraged by Peter Medawar's 1953 report (2) that immunological rejection of skin grafts exchanged between non-genetically identical mice could be abrogated by induction of transplantation tolerance and by Loutit's work showing restoration of hemopoiesis in irradiated mice given spleen cells from the same inbred strain, but not from a different strain (3).

At that time, in the 1950s, there was limited knowledge of the genetics of transplant antigens and the immune responses to them, and all of those first patients died, although transient chimerism was recorded. In 2018, 60 years later, hundreds of thousands of hemopoietic transplants have been carried out, using a variety of sources for stem and precursor cells and an array of pre-conditioning treatments to facilitate graft acceptance in patients. While many recipients survived, cured of hematological malignancies or hematological diseases that would otherwise have killed them, others suffered serious side effects of which graft-vs.-host disease (GVHD) has been the most challenging. This uncomfortable “fact of life” has limited the more widespread use of hemopoietic transplants to treat other conditions that might benefit from “resetting the immune system,” such as autoimmunity and rejection of therapeutic organ transplants.

Advances in pharmacology and the development of less toxic preconditioning regimes have made a series of stepwise improvements, both in graft acceptance and reducing GVHD incidence and severity. These have been built on advances in genetics, particularly with respect to delineation of the major histocompatibility complex (MHC), HLA in humans, along with homologs in species used in preclinical research, mice (H2), dogs (DLA), and non-human primates (SLA). In parallel, increasing knowledge of the immune system has provided insight into factors regulating the quality and quantity of immune responses, and has triggered the development of a range of biologically active pharmaceuticals aimed at controlling over- or under-effective responses in patients. The enrolment of patients into controlled clinical trials is the ultimate way to test safety and efficacy of new treatments: this is now widely embraced.

Our speakers in 1992 included those working on hematopoietic stem cells (Nydia Testa, Maria Grazia Roncarolo, Peter Hoogerbrugge) on identification of major and minor histocompatibility antigens (Robert Lechler, Elizabeth Simpson, Giovanni Ferrara), on immune responses to transplants (Herman Waldmann, Manlio Ferrarini, Yair Reisner) and on treating patients with hematopoietic disorders with HSC transplants (Jill Hows, Andrea Bacigalupo, Bruno Rotoli, Andrea Velardi, Guido Lucarelli).

## Immune Responses to Transplants

Hemopoietic stem cell transplantation (HSCT) is the forerunner of both cell and gene therapies, which depend on slipping potentially foreign components past homeostatic controls limiting cell numbers and immune responses fine-tuned by evolutionary selection for protection against pathogens. The numbers of cells comprising some tissues can be reduced by irradiation and/or cytotoxic drugs to provide “space” for the introduced cell population to settle and proliferate. The appropriate dose of space-inducing treatment depends on the tissue and on whether total replacement or chimerism is required for therapeutic effect.

However, the immune response remains a formidable barrier, comprised of a moving army of variously armed host cells along with cell-bound and shed molecules, such as antibodies, receptors and cytokines, orchestrated by a complex activatory and inhibitory pathways. For hemopoietic transplants the situation is further complicated by potential two way reactions between recipient and donor: rejection of donor cells is the host-vs.-graft (HVG) response, whereas attack of the host by cells in the donor innoculum is the graft-vs.-host (GVH) response. Graft-vs.-host disease (GVHD) occurs when normal host tissues are attacked, but when this is focused on host tumor cells, the terms graft-vs.-leukemia (GVL) or graft-vs.-tumor (GVT) are used. Separating GVHD from GVL/T has proved difficult. Though most of the target antigens are shared, in principle there could be a set of non-shared tumor antigens. Unfortunately, which patients will develop GvHD and/or GVL cannot yet be accurately predicted because the molecular targets have not been sufficiently identified. The extraordinary diversity of target antigens is amplified by HLA polymorphism as well as that of minor histocompatibility antigens and tumor antigens arising from serial mutation.

Peter Medawar demonstrated that rejection of skin grafts exchanged between genetically dissimilar rabbits or mice showed specificity and memory (4)—hallmarks previously ascribed to antibody responses against pathogens. Mitchison subsequently transferred skin graft rejection with lymphocytes, but not serum, i.e., it was cell and not antibody mediated (5). On the basis that immune responses evolved to discriminate between self and non-self, Medawar designed experiments in which cells from one inbred mouse strain were introduced to immune-incompetent pre-natal or neonatal mice of another strain to induce recognition of them as “self” during development. When skin grafted as adults, most of the injected mice showed prolonged acceptance of test grafts. These experiments were replicated in other mammalian species and in birds (2, 6). Thus, the possibility of inducing transplantation tolerance existed, giving encouragement to both hematologists like Donnall Thomas and surgeons like Joseph Murray who performed the first kidney transplants in humans.

Inducing tolerance in adult animals, either humans or experimental species, proved more difficult. Making recipients immunoincompetent using irradiation and/or cytotoxic drugs abrogates HVG but can lead to collateral damage to host tissues, and if immunocompetent lymphocytes of donor origin are included in the donor graft, they can cause GVHD. The morbidity and mortality figures during the early years of clinical BMT were daunting but they sparked extensive and focused preclinical experiments in outbred dogs (by Thomas' group) leading to step-wise improvements in the clinical protocols used, including reduced levels of irradiation and the development of less toxic drugs for pre-treatment of recipients. Pretreatment of donor cells was also trialed including removal of contaminating lymphocytes from bone marrow and use of alternative sources, such as mobilized stem cells isolated from peripheral blood or cord blood as a source.

In the 1960s and 1970s basic research studies had probed the composition of lymphocyte populations, leading to the understanding of the different functions of thymus derived (T cells) and bone marrow (B cells) lymphocytes and of T cell subsets, Th helper, and Tc cytotoxic cells. The development of monoclonal antibodies by Milstein and Kohler in 1975 (7) led to the isolation of highly specific reagents for identifying and separating cell types on the basis of cell surface molecules. Separation of cells with characteristic markers using the fluorescence activated cell sorter developed by Len Herzenberg in the 1970s and 1980s (8) was crucial to defining the phenotype and function of hemopoietic and lymphopoietic cell subsets.

Isolation from mouse bone marrow of selected populations containing a high proportion of haemopoietic stem cells (HSC) that could repopulate all lineages was shown by Weissman in the late 1980s and early 1990s (9). However, monoclonal antibodies defining the homologous population in humans have been more difficult to develop for approved clinical use. Even low levels of contaminating T lymphocytes in partially purified sources of hematopoietic stem cells can cause GVHD. If T cells are completely removed leukemic relapse is more likely, although that risk can be significantly reduced by donor lymphocyte infusion (DLI) following T cell depleted hematopoietic stem cell transplantation (10).

The molecular identification of the targets of transplant rejection (histocompatibility antigens) has played a central role in all clinical transplantation, but hematopoietic cell reconstitution presents the greatest challenges because of GVH, but also the greatest rewards, with the development of GVL/GVT effectors.

At the 1992 Ceppellini School BMT course the speakers outlined the key findings underlying the development of the field and presented them for discussion along with recent advances. It was clear that the problem of GVHD remained serious, with questions about identifying the best tolerable genetic match between donor and recipient, and how to minimize and treat this complication. Since 1992 then there have been substantial advances in knowledge of stem cell biology, the genetics of histocompatibility antigens and of the cells, molecules and regulatory circuits of the immune system, permitting further stepwise improvements, but now, having looked at the immune response, let us consider how transplantation antigen genes and their products were originally identified, especially as key research, including links with a range of human diseases, was carried out in Italy.

## Genetics, Molecular Identity and Function of Transplantation Antigens, Major and Minor

George Snell took a systematic genetic approach to enumerating and mapping loci responsible for graft rejection with experiments transplanting skin and tumor grafts between inbred mouse strains, their F1 hybrids and backcross progeny. These were carried out at the Jackson Laboratory in the late 1930s before DNA, genes or chromosomes had been identified as units of inheritance. Instead, co-inheritance of traits mapped them into so-called “linkage groups.” Snell numbered his histocompatibility loci, H1, H2, H3, etc., according to their apparent strength, with H2 the strongest, eliciting the fastest graft rejection. It was named the major histocompatibility (H) locus, with the others designated minor H loci. The agglutinating antibodies developed by Peter Gorer following immunization of different mouse strains were found to detect alleles of H2. Snell's 1948 paper (11) summarizes findings defining not only the major but also a number of minor H loci, of which more were found by Snell and his collaborators Bailey and Taylor who isolated and mapped each H locus in congenic and recombinant inbred mouse strains. However, while MHC antigens are highly polymorphic, their minor H counterparts are not. They are either di-allelic or characterized by one expressed and one non-expressed allele.

Ceppellini played a key role within the international consortium (12) in discovering the human homologs of H2 antigens, the human leukocyte antigens (HLA) named from their expression on human peripheral blood lymphocytes, to which agglutinating antibodies from multiparous women were found, directed against paternal alloantigens. Ceppellini's genetic studies in the 1950s on hemoglobinopathies, linking resistance to malaria and thalassemia, was followed by his immunogenetic research on red blood cell antigens, leading him to recommend to Dausset, then working on the elusive human leukocyte antigens using poorly reproducible agglutinating assays, the study of identical twins, whose reactions should be the same if the antigens were genetically controlled. The power of a genetic approach was used by Ceppellini when he HLA typed large families, identifying siblings inheriting the same parental HLA alleles, those with different alleles, and those with one shared allele. He then exchanged skin grafts between them and discovered while the most rapid rejection took place when no HLA alleles were shared, that for even completely HLA matched pairs graft rejection was only delayed by a week or 2 (13). These findings, in parallel with Snell's on mice, were evidence that minor H antigens also existed in humans. Confirmation of this comes from clinical bone marrow transplantation: HLA matched sibling recipients can still suffer life-threatening GVHD directed against minor H allo antigens.

A breakthrough in interpreting the human HLA antibody data accumulated during the international histocompatibility workshops came when viewed as a complex of linked loci, each with a number of alleles, rather than as a single locus. This arrangement had already been observed for the mouse MHC, H2, to consist of two linked polymorphic loci (named H2K and H2D), encoding cell surface molecules expressed on all lymphocytes in peripheral blood (PBL). Other mouse anti-H2 antibodies were found that reacted with B but not T lymphocytes. These were directed against the products of loci within H2 that distinguished alleles associated with the ability to respond to certain haptenated proteins, i.e., those were “Immune Response” (Ir) gene controlled. The Ir genes mapped between H2K and H2D of the mouse MHC complex on chromosome 17 and were named H2A and H2E. H2 studies were easier than those on HLA because of inbred mice, including congenic strains with selected alleles of H2 backcrossed onto a standard strain. Intercrossing could then be carried out to create H2 recombinants, allowing the study of individual loci. In outbred populations, such as humans HLA recombination occurs at a low frequency depending on the chromosomal distance between loci—HLA genes are closely clustered on human chromosome 6.

To resolve a nomenclature clash that had occurred between different laboratories working on human MHC, the first human HLA loci were named HLA-A and -B, and between them a third locus, HLA-C was mapped. The human homologs of mouse Ir genes mapped them just outside (centromeric) the A/B/C region and were named DR, DQ, and DP. Their cell surface molecules, like Ir genes of mice, are not expressed on all somatic cells but on human PBL they were detected on both B cells and activated T cells. Mouse Ir genes and their human homologs were designated MHC class II, to distinguish them from H2K and D, and HLA A, B, and C, that were known as MHC class I.

Understanding the functional homologies of the mouse and human MHC relied on the development of *in vitro* assays of T cell function. Proliferation assays developed by Fritz Bach identified MHC class II alleles as stimulatory in one-way mixed lymphocyte cultures (MLC) between two mis-matched individuals where cells from one were irradiated to prevent them proliferating (14). In parallel studies in other laboratories cytotoxic T cells (Tc) were developed in MLC, initially in mice (15). Tc effector cells in these cultures were directed against MHC class I antigens, while helper T cells, Th, specific for MHC class II were required for optimal responses (16). The same is true for both species.

*In vitro* cultures of lymphocytes were also used to examine the fine specificity of cytotoxic T cells against viral epitopes and minor (H) histocompatibility antigens. The paradigm changing paper on this was published in 1974 by Zinkernagel and Doherty, who showed that *in vitro* re-stimulated spleen cells from mice immune to lymphocytic choriomeningitis virus (LCMV) generated cytotoxic T cells that recognized virus only in association with self MHC, i.e., they were “MHC restricted.” The same was found to be true for mouse and human cytotoxic T cells specific for the male specific minor H antigen, HY (17, 18) and all other minor H antigens. Although the manner in which T cells recognized both self MHC and a foreign antigen was not understood until the crystal structure of HLA-A2 was solved in 1987, showing a peptide fragment of viral or other origin held in the peptide binding groove of the MHC molecule (19). The generation of minor H specific T cells and clones provided key reagents for mapping and cloning the corresponding genes and identifying their MHC binding peptides. This is also true for a tumor specific antigens (TSA): Thierry Boon led investigation of these by generating TSA specific T cell clones (20), and used these to expression clone a range of melanoma and other tumor antigen genes and explored use of them to immunize patients. This approach also informs treatment of leukemia patients, given HSC.

The *in vitro* T cell correlates of HVG and GVH immune responses against minor H antigens are MHC class II restricted Th helper cells and class I restricted Tc cytotoxic effectors. Unlike *in vitro* responses to mismatched polymorphic MHC antigens that develop in primary cultures, those to minor H antigens require previous *in vivo* exposure to antigen to increase the precursor frequency of T cells specific for the minor H antigen(s). Two MHC matched individuals in an outbred population will differ at many minor H loci, including HY in the case of brother/sister pairs. Immunodominance of the response against one or more minor H antigen occurs. An *in vivo* manifestation of this is stronger GVHD in male recipients of HLA matched female hematopoietic transplants than in female recipients, since HY is immunodominant to some other minor H antigens. However, the strength of each is also a function of the MHC restricting molecule, since it is the combination that determines immunogenicity.

The 1992 Ceppellini course on BMT included presentation and discussion of the research leading up to the discovery of both MHC and minor H antigens in humans and mice. This included methods then currently in use for HLA typing, and how MHC restriction prevailed for the recognition by T cells of all non-MHC antigens, whether minor H, viral and other.

Responses against multiple minor H antigens are strong and can be life threatening in GVHD. In contrast, if they could be separated into their components specificities they could be therapeutic, particularly for selecting those directed against minor H antigens preferentially expressed on tumor cells. An opportunity to create this situation occurs in leukemia patients given HSC transplants. Providing that expression of a minor H antigen is hematopoietic cell specific, cytotoxic T cells directed against the recipient allele of that antigen will target both leukemic cells and residual recipient hematopoietic cells but not those of repopulating donor origin. Such curative T cells would remove leukemic cells without the side effect of damaging other recipient tissues.

The treatment outlined above would require identification of minor H antigens expressed only on hematopoietic cells, for which there are some candidates, together with the isolation and expansion of effector T clones that can be approved for clinical use. Those of donor stem cell origin would be ideal, as they should persist long term in the recipient and mitigate against leukemic relapse. Current research on effector cells transduced with CAR-based or T cell receptor (TCR)-based constructs is relevant to this.

## Sources of HEMATOLOGICAL Stem Cells (HSC)

HLA typing was recognized early as crucial for allogeneic HSC transplantation, as HLA mismatched grafts were likely to be rejected and/or cause severe GVHD. Use of HLA matched sibling donors reduced but did not remove this risk which was higher when non-sibling HLA matched family donors were used. Use of unrelated HLA matched donors (MUD) increased GVHD risk above that. Haploidentical donors, i.e., those with one of their two HLA haplotypes inherited from a parent, especially the mother, was started in the 1990s and has been substantially increased as methods for abrogating or treating GVHD have improved. Such a procedure has become more common, especially after the development of conditioning regimens involving cyclophosphamide (21) that appear effective at generating the early expansion of regulatory T cells.

Haploidentical transplantation has provided a unique platform for experimental tolerogenic strategies, with several studies providing convincing evidence that, at least when using the most appropriate donor, the outcome can be very good (22). A recent retrospective study (23) has convincingly documented that it is the patient and disease rather than donor features that affect survival of these patients. However, it is important to acknowledge the fact that the technique of haploidentical transplantation exposes patients to delayed immune reconstitution thus potentially limiting some of the benefits.

The use of bone marrow cells and G-CSF mobilized bone marrow cells as sources of HSC have been modified in a number of ways in attempts to reduce GVHD. Complete removal of T cells can be effective, but leukemic patients then have higher relapse rates, due to the removal of GVL effectors. Reduction in the number of contaminating T cells can help, but is difficult to titrate. Mitigating the risk of relapse, donor lymphocyte infusions (10) have been used and these, following HSC transplantation, have provided long-term curative treatment, particularly for chronic myeloid leukemia. Since T cells in these donor inocula are long lived and likely to contain a number of different clones with specificity for several transplantation antigens, mutant leukemic cells are likely to be targeted as they arise, a situation not replicated when targeted molecular therapy is given, directed against a determinant whose expression can be downregulated by mutation.

The use of cord blood as a source of HSC uncontaminated by primed T cells is practical only for child recipients, as single donations rarely contain sufficient stem cells to achieve engraftment. However, recent data suggests the opportunity to use aryl hydrocarbon antagonists to produce a robust expansion of hematopoietic stem and progenitor cells (24).

Autologous HSC avoided the risk of GVH and HVG reactions but its use in treatment of leukemias and other cancers was bedeviled by high relapse rates, as there can be no GVT effect. However, since the early 2000s the introduction of somatic gene therapy for inherited immunodeficiencies has been made possible by the identification of some of the relevant mutant genes, and methods for transducing corrected copies of them into autologous HSC *ex vivo* before transplanting them into the patient pre-treated to provide “space” for the newcomers. There are still issues that can limit the applicability of the gene therapy approach. On one side the modification of HSCs may reduce their capacity to engraft, whilst on the other the modification strategy may require the selection of the gene-corrected cells, thus impacting on the cell yield required to be efficiently transplanted (25).

Based on the notion that it is the graft-vs.-tumor effect that secures long-term eradication of the underlying malignancy, the conditioning regimens used to prepare patients for transplantation have been radically revisited. Whilst radiation was the main component of the pre-transplant conditioning because of its efficacy in eliminating replicating cells, other milder approaches have been used since the end of the 90s. Chemotherapeutic agents are now being used at doses by far lower than before, thus reducing toxicity and eventually reducing the frequency of GvHD that is largely affected, not only by the transplantation antigens but also and perhaps more importantly, by the cytokine storm induced by the tissue damage consequential to chemo/radiotherapy (26). Furthermore, it was shown that the use of cyclophosphamide soon after HSC infusion could mitigate the incidence of GvHD by increasing the number of regulatory T cells (27).

Unfortunately, GvHD remains the most dreadful complication of allografting and when refractory to steroid treatment the associated mortality is dismal. Cellular therapies may provide an alternative to traditional immunosuppressive approaches because they may provide an immunological reprogramming of the patient's inflammatory environment. Important milestones in this direction have been provided by the use of regulatory T cells (28) and mesenchymal stromal cells (MSC). Initially identified as tout-court immunosuppressants (29), MSC have been recently shown as effective at reprogramming the recipient phagocytic system to control unwanted inflammation (30). This has transplanted into very encouraging clinical experience (31).

## HEMATOLOGICAL and other Disease candidates for HSC Transplants

Leukemias, bone marrow failures, hemoglobinopathies (e.g., thalassemia, sickle cell disease) and immunodeficiencies have been mentioned, and advances with these could lead to HSC transplant-based treatments for solid tumors and other genetic diseases (e.g., lysosomal storage disease) as well as autoimmunity.

Reducing the incidence and severity of GVHD following HSC transplant remains the biggest challenge for both existing patients and the possibility of extending this treatment to additional diseases.

## Author Contributions

ES and FD drafted the review together. ES concentrating on the historical background and FD on the clinical aspects.

### Conflict of Interest Statement

The authors declare that the research was conducted in the absence of any commercial or financial relationships that could be construed as a potential conflict of interest.
